# Harnessing the gut microbiome: a potential biomarker for wild animal welfare

**DOI:** 10.3389/fvets.2024.1474028

**Published:** 2024-10-02

**Authors:** Sam Sonnega, Michael J. Sheriff

**Affiliations:** Department of Biology, UMass Dartmouth, Dartmouth, MA, United States

**Keywords:** welfare, gut microbiome, ecology and behavior, stress, wildlife

## Abstract

The welfare of wild animal populations is critically important to conservation, with profound implications for ecosystem health, biodiversity, and zoonotic disease transmission. Animal welfare is typically defined as the accumulated affective mental state of an animal over a particular time period. However, the assessment of animal welfare in the wild poses unique challenges, primarily due to the lack of universally applicable biomarkers. This perspective explores the potential role of the gut microbiome, a dynamic and non-invasive biomarker, as a novel avenue for evaluating animal welfare in wild animals. The gut microbiome, through interactions with the host’s physiology, behavior, and cognition, offers a promising opportunity to gain insights into the well-being of animals. In this synthesis, we discuss the distinction between fitness and welfare, the complexities of assessing welfare in wild populations, and the linkages between the gut microbiome and aspects of animal welfare such as behavior and cognition. We lastly elucidate how the gut microbiome could serve as a valuable tool for wildlife managers, with the potential to serve as a non-invasive yet informative window into the welfare of wild animals. As this nascent field evolves, it presents unique opportunities to enhance our understanding of the well-being of wild animals and to contribute to the preservation of ecosystems, biodiversity, and human health.

## Introduction

Animal welfare is of paramount global importance, as it directly impacts biodiversity, ecosystem health, and the ethical treatment of animals (1). Though the concept originated largely in captive and domestic contexts, researchers have recently noted that it is critical to consider the welfare of wild populations (2). Measuring wild animal welfare, both of individuals and of populations, may give managers new insights into optimizing ecosystem function. Despite its significance, there remains a notable lack of reliable physiological biomarkers for accurately assessing animal welfare, particularly in wild populations (3). Emerging research highlights the gut microbiome as a promising tool in this regard, given its profound influence on host physiology and behavior (4–7).

In this perspective, we explore the intersection of the gut microbiome and animal welfare, first discussing animal welfare and its relationship to *fitness.* Then, examining current welfare assessment methods, the role of stress and behavior, and the potential of the gut microbiome to serve as a novel biomarker. Through a brief review of existing evidence, we aim to outline the potential of the gut microbiome as an important indicator of wild animal welfare, with the overarching goal of advancing our understanding of how this novel biomarker can be harnessed to promote the welfare and conservation of wild species. Additionally, we will confront the challenges and limitations inherent in this endeavor and propose directions for future research in this burgeoning field.

## Animal welfare

Animal welfare is as challenging to define as human welfare, because it ultimately hinges on the definition of subjective internal states. Nonetheless, there have been many rigorous attempts to make welfare a measurable empirical outcome. Animal welfare can be defined most broadly as a state in which an animal is ‘fit and feeling good’ (8). We define welfare here as the affective states experienced by an individual over a given time period (3). While this is a highly subjective definition fundamentally inaccessible to outside observers, there are observable metrics of an animal’s coping capacity measuring how an animal responds to perturbations (9) that may help to triangulate this internal affective state. More specifically, the five domains model of animal welfare proposes that researchers assess nutritional, environmental, health, behavioral, and mental states of an animal to arrive at what might be called the welfare state of an individual (1). Since its introduction in 1994, the five domains model has almost exclusively been applied in agricultural, veterinary, and zoo contexts (10). Recently, interest has turned to applying models of welfare assessment to non-captive wild populations (11). Understanding wild animal welfare may provide predictive insights into wild animal fitness, as is the definition of welfare.

### Welfare vs. fitness

Ecologists and evolutionary biologists are most often concerned with fitness, here referring to an individual’s ability to survive and reproduce (12). Yet, understanding individual welfare may provide valuable insights into ecological stressors and challenges animals may face, which can directly impact their fitness. While fitness and welfare have sometimes been conflated, it is important to understand their relationship given that at times they may be positively correlated, but at others times, may not. For example, chronically elevated predation risk has been shown to reduce reproduction (13, 14) and also negatively impact animal welfare via physiological (15, 16), nutritional (17, 18), behavioral (19, 20), and mental states (21, 22) of wild animals. Conversely, there is a wealth of literature demonstrating that aspects of reproduction, like gestation and lactation, incur costs to the individual (23) and that an increase in reproductive output is often correlated with a decrease in individual body condition (24). In many cervids, for example, the association between female reproductive success and indices of body condition change seasonally, where poor body condition is associated with high reproductive output in the spring largely due to costs of lactation (25), but is then associated with decreased survival through winter (26). In cases of terminal investment, reproductive output may increase as a ‘last-ditch’ effort near the end of an individual’s life, despite decreases in body condition (27). For example, Creighton et al. (28) reared burying beetles (*Nicrophorus orbicollis*) on small vertebrate carcasses and found that older females allocated fewer resources to their own body condition and instead produced larger broods compared to younger females. In this case, a rough proxy of welfare (body condition) and fitness are inversely correlated. Thus, these relationships can be complicated and depend upon the timing of measurements. While at times there may be a disconnect between animal welfare and fitness, in understanding the relationship between the two an individual’s welfare may provide a powerful predictive tool for wildlife biologists and managers enabling proactive management strategies.

## Current assessment of welfare

Assessing animal welfare is a multidimensional and challenging task, further compounded by the dynamic complexity of wild populations (29). It relates to the extent to which the animals’ needs and preferences are met, ensuring that they are free from unnecessary suffering and capable of expressing natural behaviors (30). In the wild, assessing animal welfare presents unique challenges due to the stochastic nature of natural environments and the dynamic responses animals have to these challenges. Nonetheless, understanding and prioritizing animal welfare in wild populations is of paramount importance, as it not only relates to the ethical treatment of individual animals but also has broad-reaching consequences for biodiversity, ecosystem health, and the potential transmission of zoonotic diseases to humans (31). The lack of consensus on objective welfare assessment methods for *captive* animals (29) has long been a point of contention within the communities of both science and philosophy and this is even further exacerbated when evaluating animals in their more complex natural habitats (32). Biologists concerned with wild animal welfare often turn to more simple behavioral and physiological biomarkers, such as stress hormones and their downstream metabolites, whose ease of sampling and interpretation are alluring in the face of such complexity (33, 34).

### Behavior and welfare

Observing and interpreting the behavior of animals is a commonly employed method in welfare assessment, and is the 4th of the 5 domains of animal welfare conceptualized by Mellor et al. (1). Behavioral indicators, such as activity levels, vocalizations, and aggression, can provide insights into the emotional and physical state of animals. However, these indicators are often context-dependent and may not be universally applicable to all species and situations (35). Traditional methods of behavior assessment have been developed and refined in controlled environments, such as veterinary clinics, laboratories, zoos, and agricultural production (10). These settings often employ well-established protocols for assessing behavior through observations, observer questionnaires, or structured experiments (8). Commonly used metrics include activity levels, social interactions, vocalizations, and stereotypic behaviors (36). In recent years, novel techniques have been developed to assess animal welfare through the observation of facial expressions, body postures, and other non-invasive indicators. The Grimace Scale, for example, is a standardized tool for evaluating pain and distress in mammals based on facial expressions (37). While these approaches provide valuable insights into animal behavior in captivity, translating them to the wild can be challenging, as they may not encompass the full spectrum of behaviors exhibited in natural settings (11).

Behavior assessment techniques tailored for wild animals are emerging as vital tools for welfare assessment. These include the use of personality tests, such as measuring boldness and exploration behaviors, which can offer insights into individual variation and stress responses in natural environments (38). Behavioral ecology studies, involving the observation of foraging, mating, and predator avoidance behaviors, may also contribute to our understanding of the well-being of wild animals (39). For example, Mell et al. (40) found that personality was correlated with both metabolic expenditure and glucocorticoid stress response in common lizards. Researchers are also exploring ways to monitor behavior non-invasively through remote sensing technologies, including camera traps, acoustic recorders, and GPS tracking (41, 42). These innovative tools enable the observation of animal behavior while minimizing human interference, making them particularly valuable for assessing welfare non-invasively and in remote or sensitive ecosystems. While promising, behavioral metrics will still require validation through other welfare-related metrics, such as stress physiology. Despite these potential advances, behavior remains only one aspect of the totality of an individual’s welfare state, and linking external changes in behavior to internal changes in an animal’s physiology is critical.

### Stress physiology and welfare

Stress, in particular the pathological results of being exposed to chronic stressors, is an essential component of welfare assessment, given its direct connection with the overall well-being of animals (43). Stressors can manifest in various forms, including environmental changes, predation pressures, food scarcity, and interactions with conspecifics or other species. However, the duration and intensity of the stressor is critical to consider when potentially extrapolating stress metrics to welfare states. The stress response of vertebrates is highly conserved, and functions broadly to aid the organism in maintaining homeostasis in the face of a wide array of ecological challenges (44). The hypothalamic pituitary adrenal (HPA) axis serves to regulate the secretion of key adrenal hormones, namely glucocorticoids, involved in maintaining this homeostasis. Due to the pleiotropic nature of HPA-associated hormones (45) and their relative ease of sampling, they have been widely studied (46). The most common glucocorticoid in mammals, cortisol, is a critical mediator of energy balance and has downstream effects on numerous cell types and physiological systems (34). However, interpretations of stress hormone concentrations in the context of animal welfare are not always straightforward. Romero and Wikelski (47), for example, found that marine iguanas (*Amblyrynchus cristatus*) exposed to tourism had *lower* glucocorticoid concentrations than more isolated animals. In the context of animal welfare, these results are difficult to interpret. It is equally possible that tourist-exposed iguanas are habituated, and are therefore in a more positive welfare state relative to undisturbed animals, *or* that tourist-exposed iguanas are displaying a degraded HPA response, and a more negative welfare state. It is also important to note that the glucocorticoid response is a non-specific response, triggered by a broad array of internal and external perturbations (48). Thus, a clear understanding of the stressors involved and an individual’s response to each is necessary for the appropriate use of stress hormones as an indicator of individual welfare. Beyond glucocorticoids, stress is commonly measured using a variety of peripheral downstream markers such as free fatty acids, glucose, neutrophil to lymphocyte ratio (15), oxidative status (49), and leucocyte coping capacity (50). While many of these metrics involve invasive sampling methods (capturing the animal and drawing blood), some metabolites may be sampled non-invasively (e.g., from feces, hair, or saliva) (34). However, while non-invasive sampling methods are beneficial from a cost and welfare perspective, they may be more difficult to interpret given the increased variability due to individual differences in metabolism, differing rates of degradation, and uncertainty of the temporal scale of integration (51).Thus, validation is critical. Similar to behavioral assessments of welfare, linking changes in stress-related metrics to other physiological changes, such as the gut microbiome, may provide a more holistic understanding of an individual’s welfare.

## The gut microbiome

The microbiome refers collectively to the assemblage of microorganisms (bacteria, archaea, virus, fungi, protozoans, etc.) that colonizes various regions of an organism (52), and whose cells may outnumber host cells by an order of magnitude (53). Usage of the term microbiome is also commonly extended to include the broad array of metabolites and structural molecules (proteins, lipids, etc.) produced by microbial communities, many of which have direct effects on the host, such as impacts on the immune system (54), digestion (55), development (56, 57) and even behavior (58). These phenotypic changes in the host may then feedback to have an effect on the microbiome (59). While “microbiome” and “microbiota” are frequently used interchangeably, only “microbiota” refers to the community of microorganisms (independent of the host environment and genetic or metabolic biproducts). However, for the purpose of this article, we will follow convention, and simply use “microbiome” when referring to the microbial community.

The microbiome represents a highly dynamic system, associated with host physiology and behavior, and is critical to understanding changes in host fitness (60). Recent literature has focused primarily on the gut microbiome, as this region has perhaps the most direct interface with the host (61). Typically, the microbiome is characterized by taking a genetic inventory through extracting microbial genetic material from fecal samples or gut biopsies, and amplifying and sequencing a taxonomic marker gene. Typically, the 16 s RNA gene is used for bacteria and archaea. Bioinformatic methods can then be applied to infer the *relative* abundances of operational taxonomic units (OTUs) within the complex microbial community. Absolute abundances of microbial groups (‘microbial load’), however, can vary markedly across individuals (52). An approach known as quantitative microbiome profiling (QMP) accounts for the potential variation in individual microbial load and may yield different answers than the standard proportional approach (62). Lastly, additional functional analyses such as metagenome sequencing (63) and metabolomic analysis (64) have the potential to provide direct mechanistic insight into the broad array of metabolites which may modulate host physiology and behavior.

### The microbiome as a possible tool for welfare assessment in the wild

In recent years, there has been a burgeoning interest in investigating the gut microbiome’s potential as a novel biomarker for assessing the welfare of animals (7). Aspects of the gut microbiome and its interaction with the host may serve as a reflection of an animal’s health, stress levels, and emotional states (65). For example, Kraimi et al. (66) showed that germ-free Japanese quails (*Coturnix japonica*) displayed decreased emotional reactivity across a range of tests including tonic immobility and novel-object recognition. Applied to wild populations, the intersection of the gut microbiome with animal welfare opens the door to a promising avenue of research with implications for conservation, wildlife management, and the broader field of ecology. In addition to its profound effects on the physiology and behavior of its host, the gut microbiome is a primary candidate to measure animal welfare given its ability to be sampled via fecal collection. One of the major challenges in measuring welfare in wild animals is to ensure the measurements themselves do not reduce welfare, and as such, the use of a non-invasive method is preferred. Thus, the gut microbiome has the potential to serve as a non-invasive yet informative window into the welfare of wild animals (67, 68).

### The gut microbiome and individual physiology and behavior

The gut microbiome holds promise as an estimator of individual welfare given it is in constant dialogue with many aspects of host physiology, including energy metabolism (69), thermal regulation (70), fat deposition (71), immune function (72), and endocrine regulation [reviewed in Williams et al. (73)], and its influence on host behavior (74, 75). A primary pathway through which behavior is affected, both directly and indirectly, is via the central nervous system of vertebrates in what has become known as the ‘gut-brain axis’ (4). For example, in a landmark study Sudo et al. (76) demonstrated the importance of the gut microbiome in the development of a functional stress response in lab mice. They found that germ-free mice exhibited hyper-sensitive stress responses, elevated levels of corticotropin-releasing factor (CRF) mRNA in the hypothalamus, and decreased expression of brain-derived neurotrophic factor (BDNF) in the cortex and hypothalamus. These effects were reversed when mice were re-colonized with a fecal transplant from a normal donor. In another welfare-related study, adult zebrafish supplemented with *Lactobacillus plantarum* displayed attenuated anxiety-related behaviors and altered serotonergic and GABAnergic signaling pathways in the brain. Given that many behaviors influenced by the microbiome in laboratory systems are critical for the well-being and adaptive success of wild populations (59, 77), the microbiome is likely to play a significant role in wild animal welfare. Despite its centrality to the physiology of welfare states, there have been few ecological studies specifically investigating the gut-brain axis in relation to the welfare of wild animals. Taken together, the gut microbiome has clear associations with the physiology and behavior of the individual, making it a potential bridge between environmental stressors, the physiological and behavioral responses of the individual, and the expression of welfare-related states. It is critical, however, to validate the links between welfare states and aspects of the gut microbiome before using it as a metric to assess the welfare of wild animals or populations (78). An important caveat when considering the usage of the gut microbiome as a welfare biomarker is that the relationship between the microbiome and welfare states is bidirectional, with microbiome changes potentially both influencing and reflecting welfare states. The directionality of this relationship is likely to be dependent on the time over which aggregate welfare is being measured, and the specific changes in microbial composition. This complexity underscores the need for further research into the dynamics of state-behavior feedback loops (79) as they relate to gut microbial communities.

### Implications for the management of populations

Assessment of the gut microbiome holds significant promise for wildlife managers who are tasked with monitoring and conserving biodiversity, given its relationship with individual welfare and links to reproductive success. For example, stressor-induced alterations in the gut microbiome (80, 81) can reduce an animal’s reproductive success (82). Conversely, it has been shown that the administration of a probiotic can increase ovarian function and oocyte maturation (56). In addition to its role in physiological and behavioral changes, the gut microbiome’s role in modulating immunity and health can also have implications for the reproductive success of individuals (73). Thus, by monitoring the gut microbiome, wildlife managers can gain valuable insights into the overall health and stress levels of animals, and potential for reproductive fitness. This information can inform conservation strategies, allowing for the timely identification of populations at risk and the implementation of targeted interventions to mitigate threats (83). While current research on the microbiome is insufficient to support its immediate implementation as a management tool, ongoing studies may illuminate its potential. Wildlife managers could eventually employ non-invasive microbiome assessments to monitor health and stress in populations, helping to identify at-risk populations and guide conservation strategies.

Conclusion and future directions

While the potential role of the gut microbiome as an indicator of animal welfare in wild populations presents exciting prospects, it also underscores the need for further research and exploration into the complex relationships between aspects of welfare and changes to host microbial communities in wild animals. Here, we outline several key avenues for future research:

1. Species-Specific Considerations: The gut microbiome is highly variable across different animal species (6, 84, 85). Future research should focus on characterizing the gut microbiomes of a wide range of wild species and explore how species-specific differences in microbiota composition and function relate to their welfare. Such research could unveil unique biomarkers for various taxa and ecosystems.
2. Ecological Context: Wild animals exist within complex ecosystems, and their well-being is intricately linked to the health of these systems. Future research should consider how environmental factors, including habitat quality, food availability, and predation pressure, interact with the gut microbiome and impact animal welfare. Understanding these ecological dynamics is critical for effective conservation efforts.
3. Temporal Dynamics: Long-term studies are essential to uncover the temporal dynamics of the gut microbiome and its associations with animal welfare (86). Investigating how microbiota composition and function change over seasons, years, or across different life stages can provide valuable insights into the flexibility and adaptability of this biomarker (87).
4. Integration of Multi-Omics Approaches: The gut microbiome is just one piece of the puzzle. Combining metagenomic, metatranscriptomic, and metabolomic approaches can provide a holistic view of the gut microbiome’s functional capacity and its interactions with the host (88). This integrative approach can offer a more comprehensive understanding of how the gut microbiome influences animal welfare.
5. Non-Invasive Monitoring Techniques and Validation: Developing and refining non-invasive techniques for monitoring the gut microbiome in the wild is crucial. Remote sampling methods like fecal collection should be optimized and validated for use across diverse ecosystems and species, ensuring minimal disturbance to the animals. To establish the gut microbiome as a reliable welfare indicator, its associations with stress, behavior, and health must be rigorously validated in various wild species. These associations should be quantified and tested across different contexts and environmental conditions. Additionally, researchers should take into account rates of sample degradation that may depend on age of sample, weather conditions, and temperature.
6. Comparative Studies: Comparative studies between captive and wild populations can provide insights into the impact of captivity on the gut microbiome and welfare. Understanding how captive and wild environments influence the gut microbiome and related welfare indicators is essential for managing captive populations and for assessing the welfare of released animals. Additionally, “healthy” microbiomes are likely to be context specific. An enterotype correlated to positive welfare states in the lab may not correspond to positive welfare states in wild environments.
7. Conservation Applications: The translation of gut microbiome research into practical conservation applications is an important frontier. Identifying early warning signs of population decline or health threats using the gut microbiome can facilitate timely intervention and targeted conservation efforts.

The well-being of animals in their natural habitats bears substantial significance, extending beyond individual animals to encompass broader consequences for ecosystem health, biodiversity, and the potential transmission of zoonotic diseases to humans. However, assessing animal welfare in the wild is a challenge marked by the lack of universally applicable biomarkers, emphasizing the need for innovative approaches. The gut microbiome, with its dynamic role in mediating stress, behavior, and health, and its relationship to fitness has emerged as a promising candidate for addressing this gap. By providing insights into the physiology and behavior of animals without invasive sampling, it offers a window into their physical and emotional states. In light of the multifaceted relationships between the gut microbiome, stress, and behavior, the potential to employ this biomarker for the welfare assessment of wild populations holds vast promise. Moreover, conservation biology is undergoing a paradigm shift towards wildlife management that proactively prevents chronic stress and mitigates welfare-related issues (89). In this holistic perspective, the gut microbiome becomes a valuable tool for advancing our understanding of animal welfare in the wild and enhancing wildlife management strategies. As the field continues to evolve, both conservation and animal welfare scientists should collaborate to facilitate a more rigorous understanding of what constitutes a healthy ecosystem.

## Data Availability

The original contributions presented in the study are included in the article/supplementary material, further inquiries can be directed to the corresponding authors.
